# Why confronting positionality matters to advancing trans research and discourse

**DOI:** 10.1016/j.lana.2023.100568

**Published:** 2023-08-03

**Authors:** Arjee Javellana Restar

**Affiliations:** a Department of Epidemiology, University of Washington School of Public Health, Seattle, WA, USA; b Department of Social and Behavioral Sciences, Yale University School of Public Health, New Haven, CT, USA

Trans health has become a groundswell for discussing viewpoints and research methodologies among medical and public health communities in recent years—with some trans-led discussions that have been successful in advancing science and community strategies through constructive feedback, particularly on methodologies.^1^^,^^2^ However, some dialogues derail the objective that medical and public health professionals working in trans health seek to advance, and undermine efforts to advance equity, particularly in gender-affirming care. While it is important to invite interested medical and public health professionals into the field of trans health, it is vital for them to disclose their positionality and depth of expertise in relation to the body of scientific evidence in trans health to understand potential biases.^3^

For example, in the case of the correspondent, Armitage, when replying to Restar's ‘Gender-affirming care is preventative care,^4^” displayed a valiant effort in identifying common methodological limitations, some of which are inherent to the study designs and are acknowledged within the original studies including those of cross-sectional designs (e.g., associations vs. causations), pre-post prospective cohort studies (e.g., comparison groups, period vs. lifetime measurements), and systematic reviews (e.g., risk of bias). *While critiquing study designs was not the focus of Restar's article*, Armitage took the opportunity to re-emphasize that evidence between gender-affirming care and mental health outcomes are indeed significant associations, just as Restar noted.

Armitage also proposed the need for a “comparison group of individuals who had sought but did not yet receive GAC” however, this suggestion was already considered by the original authors as inappropriate due to their study data structure and research aims.^5^ The authors of the original study remain conclusive that their findings of “longitudinal association between gender-affirming surgery and reduced likelihood of mental health treatment lend support to the decision to provide gender-affirming surgeries to transgender individuals who seek them,” and provide “support for policies that ensure coverage of gender-affirming care.”

Armitage also points out measurement tools vary between studies, which, as Restar also argues there is a critical need for better mental health screening tools for trans people. Certainly, studies that are using period measures of suicide ideation compared to lifetime measures (e.g., past year vs. lifetime) are more valuable to researchers given event recency—and particularly because measuring lifetime events are already *inclusive of* events that occurred recently. As such, studies like Chen^6^ that report lower suicidal attempts in the past 2 years and achieve significant results in that timeline, as compared to lifetime, are actually considered positive study outcomes. From an intervention perspective, it is illogical to measure and intervene in outcomes that measure lifetime events. Instead, suicide outcomes with more proximal measures are more meaningful, particularly since we are in a public health crisis in addressing trans people's suicidality, particularly trans youth.

Most importantly, Armitage's assessment of the evidence in gender-affirming care is too biased and determined—a clear demonstration of their superficial knowledge to evaluate systematic reviews. By only focusing on and recapping methodological flaws, which the original authors have clearly stated,^7^^,^^8^ it is apparent that they either did not read the actual articles or does not have the statistical knowledge to evaluate empirical research. They neglected to account that *not* a single piece of evidence across all study designs has pointed out that gender-affirming care is linked to increasing negative mental health outcomes—a classic display of statistical naivety and their depth of relationship to trans health literature.

If we, as global communities of medical and public health professionals, are to engage in trans health literature, learning our positionality both for ourselves and in relation to the work provides us with the ability to contribute meaningfully to the discourse and advance the science of trans health—instead of being a tool to parroting ‘low-quality’ arguments rooted in lack of expertise, misinformation, and biases that are evidently not in the service of trans populations' health and lives. As trans scientists, stakeholders, and community investigators along with cis researchers are engaging and leading the pathways in envisioning the future of gender-affirming care across many scientific areas and disciplines, a critical first step to such co-learning opportunities is acknowledging how researchers' positionality including their knowledge, is limited in this area—and to defer to the experts in the room, particularly those with lived experiences.

## Contributors

Dr. Restar conceptualised and wrote this correspondence.

## Declaration of interests

The author declares no competing interest.
